# One-plasmid double-expression system for preparation of MS2 virus-like particles packaging SARS-CoV-2 RNA

**DOI:** 10.3389/fcimb.2023.1238543

**Published:** 2023-11-29

**Authors:** Lili Qi, Zheng Zhang, Mengting Wang, Zhijian Ke, Haiguang Mao, Gang Deng, Jinbo Wang

**Affiliations:** ^1^ School of Biological and Chemical Engineering, NingboTech University, Ningbo, Zhejiang, China; ^2^ Blood Transfusion Research Institute, Ningbo Central Blood Station, Ningbo, Zhejiang, China

**Keywords:** MS2 virus -like particles, SARS-CoV-2, RT–qPCR, COVID-19, MS2 VLPs

## Abstract

COVID-19 is a disease caused by a virus named SARS-CoV-2. SARS-CoV-2 is a single-stranded positive-sense RNA virus. Reverse transcription quantitative PCR (RT–qPCR) assays are the gold standard molecular test for detection of RNA viruses. The aim of this study was to construct an RNA-positive control based on MS2 phage-like particles (MS2 VLPs) to detect SARS-CoV-2 RNA. pCDFDuet-1 was used as a one-plasmid double-expression system to construct MS2 VLPs containing ssRNA of SARS-CoV-2. The sequence encoding one copy of maturase, His-tag and coat protein dimer was cloned and inserted into MCS1 of the plasmid; the fragment encoding protein N and ORF1ab from SARS-CoV-2 was cloned and inserted into MCS2. The prepared plasmid was transformed into Escherichia coli strain BL2 (DE3), and expression of the construct was induced by 1 mM isopropyl-L-thio-D-galactopyranoside (IPTG) at 30°C for 12 hours. MS2 VLPs were purified and collected with Ni-NTA affinity chromatography columns. The size and shape of the MS2 VLPs were verified by transmission electron microscopy, and the stability of MS2 VLP packaged RNA was evaluated by treatment with RNase A. Effects of storage temperature and buffer on MS2 VLP stability were also investigated. The results showed that SARS-CoV-2 MS2 VLPs could be successfully produced by this one-plasmid double-expression system. MS2 VLPs showed high stability and may be used as a positive control in molecular diagnosis of COVID-19.

## 1 Introduction

Although several vaccines have been developed and distributed in response to the spread of severe acute respiratory syndrome coronavirus (SARS-CoV)-2, coronavirus infections remain a severe threat to human health. The number of COVID-19 cases will again climb as immunity from vaccines and infections wanes. It has been reported that in China, an infection cycle will occur every six months (Ye, 2023), and repeated infections might lead to health problems such as long COVID. COVID-19 is not only a pulmonary disease but can also affect almost all other organ systems (Huang et al., 2020). Previous studies had found that aging decreased the effectiveness of the immune system against pathogens. Immunosenescence causes the weakening of the immune system in people with increasing age (Zinatizadeh et al., 2023). In immunocompromised patients, the immunogenicity of COVID-19 vaccines was decreased. There was a proportion of population showed delayed and blunted immune responses to COVID-19 vaccination (El Karoui and De Vriese, 2022). Vulnerable people, such as older adults, the immunocompromised and those not responding to vaccinations are still at risk of becoming very ill.

SARS-CoV-2 is a positive-sense single-stranded RNA (ssRNA) virus (Holshue et al., 2020; Wiersinga et al., 2020). For detection of SARS-CoV-2 and other coronaviruses that can cause serious diseases, ribonucleic acid sequence testing based on RT–qPCR is an essential method (Corman et al., 2020; Pfefferle et al., 2020). However, false-positive or false-negative results are often reported with RT–qPCR detection (Afzal, 2020; Hellou et al., 2021), and such false results may result from factors such as interfering substances in RT–qPCR. To avoid false-negative results, an appropriate positive control should be used in every run of an RT–qPCR assay (Hellou et al., 2021).

MS2 is a type of positive-sense ssRNA virus with an icosahedral capsid, which is built from one copy of maturase (also called the A protein) and 178 copies of coat protein (Prakash and Gosavi, 2021). Assembly of MS2 particles is triggered by interaction of coat proteins with a stem–loop structure (called the “pac” site) in ssRNA (Dinh and Müller, 2021). The interaction can result in a sequence-specific self-assembly mechanism of maturase and coat protein. Virus-like particles (VLPs) are protein complexes that resemble a native virus capsid (Stockley et al., 2016; Dai et al., 2017; Garmann et al., 2019); they represent gene-free protein particles that are similar to natural virus particles. RNA can be encapsulated by VLPs, which are greatly resistant to degradation by RNase (Dinh and Müller, 2021). Different methods have been developed to produce MS2 VLPs carrying ssRNA molecules. In general, MS2 VLPs can be used as a positive control virus for detection of ssRNA viruses such as human Zika virus (Lin et al., 2017), norovirus (Hennechart-Collette et al., 2014), Ebola virus (Song et al., 2011), human enterovirus (EV) (Wang et al., 2016), hepatitis E virus (HEV) (Wang GJ. et al., 2015) and dengue virus (Fu et al., 2017). However, each of these systems has various drawbacks, e.g., lower assembly efficiency, complicated purification, and/or instability during storage. Accordingly, a one-plasmid double-expression packaging system has been developed to avoid such limitations. As a double-expression system, pACYCDuet-1 has been used to produce intact His-tagged MS2 VLPs. This system exhibit high efficiency with regard to expression and purification (Mikel et al., 2017). Overall, the stability of the positive control ssRNA is very important for accurate and reliable diagnostic testing for COVID-19. Furthermore, suitable storage conditions are essential for preserving the quality and longevity of MS2 VLPs. However, storage conditions such as temperature and buffer have not been explored. In this work, the pCDFDuet-1 plasmid was used to develop a double-expression system of His-tagged MS2 VLPs that carry an 1857-nt-long control sequence of SARS-CoV-2. The storage stability at -20°C and 37°C was examined, as were the protective effects of Tris-NaNO_3_ solution as a storage buffer.

## 2 Materials and methods

### 2.1 Materials

*Escherichia coli* BL21 (DE3) was obtained from TransGen Biotech Co., Ltd. Viral RNA extraction kits were purchased from Omega Bio-Tek Co., Ltd., and peptone, yeast powder and agar powder were purchased from Beijing Solarbio Biotechnology Co., Ltd. In addition, Isopropyl-β-D-thiogalactopyranoside (IPTG) and limidazole were supplied by Sigma–Aldrich, and Ni-NTA agarose resin was obtained from Thermo Fisher. Real-time PCR kits were purchased from TOYOBO Life Science Co., Ltd.

### 2.2 Construction of the plasmid pCDFDuet-MS2-SARS-CoV-2

A 2003 bp DNA sequence encoding one copy of maturase and two copies of the coat protein with His-tag modification was synthesized by Genscript Biotech Co., Ltd. The pCDFDuet-1 plasmid was treated with NcoI and NotI at 37°C for 2 hours in a 50 µL reaction mixture containing 500 ng of plasmid DNA, 5 µL of buffer solution, and 10 U each of endonucleases. The cleaved vector DNA was purified with a Gel Extraction Kit (Omega). Ligation of the fragment encoding maturase and coat protein with MCS1 of pCDFDuet-1 was performed with a One Step Cloning Kit (Vazyme) according to the manufacturer’s instructions. Then, the recombinant plasmid (pCDFDuet-MS2) was transformed into *E*. *coli* BL21(DE3). The bacteria were grown overnight on LB agar plates containing streptomycin (10 µg mL^-1^).

To meet a variety of different targeted amplicons of RT–qPCR methods for detecting SARS-CoV-2, the coding sequence of protein N and ORF1ab from SARS-CoV-2 was used as the positive control sequence. The chimeric sequence is 1857 nt in length, with one C-variant pac site of MS2 at the 3’-end. The sequence of the C-variant pac site of MS2 is ACATGAGGATCACCCATGT. The chimeric sequence was *de novo* synthesized by Genscript Biotech Co., Ltd. The pCDFDuet-MS2 plasmid was then digested by NdeI and AvrII, and the cleaved plasmid DNA was purified with a Gel Extraction Kit (Omega). Ligation of the positive control sequence to MCS2 of the cleaved pCDFDuet-MS2 vector was performed with a One Step Cloning Kit (Vazyme) according to the manufacturer’s instructions. Positive clones were selected by DNA sequencing. After sequencing identification, the confirmed recombinant pCDFDuet-MS2-SARS-CoV-2 plasmid was extracted, purified and transformed into *E*. *coli* BL21(DE3) to screen for the recombinant strain at a concentration of 50 μg mL^-1^ streptomycin on LB plates.

### 2.3 Induction of expression of pCDFDuet-MS2-SARS-CoV-2 in E. coli BL21(DE3)

The engineered *E. coli* BL21(DE3) cells harboring the pCDFDuet-MS2-SARS-CoV-2 plasmid were cultured in LB broth medium containing 10 μg mL^−1^ streptomycin at 37°C for 4 hours. Cultures of the recombinant BL21(DE3) cells were diluted 1:100 in fresh 100 mL LB medium. When the optical density (OD) 600 reached 0.4–0.6, isopropyl-*β*-d-thiogalactoside (IPTG) was added to a final concentration of 1 mM, and the culture was incubated at 30°C for 12 hours. The cell suspension was centrifuged at 10000 rpm for 10 minutes at 4°C, and then the pellet was washed three times with PBS.

The pellets were resuspended in PBS and then sonicated. To eliminate cell debris, the bacterial suspension was briefly centrifuged at 10000×g for 15 minutes at room temperature. Twenty microliters of the supernatant was mixed with 5 μL of 5×SDS sample buffer, and 15 μL of this mixture was separated by 12.5% SDS–PAGE electrophoresis.

### 2.4 Preparation and purification of MS2 VLPs containing SARS-CoV-2 RNA fragments

The supernatant from the above step was mixed with equilibrium buffer (100 mM PBS, 20 mM imidazole, pH = 8.0) for purification of the His-tagged MS2 VLPs with a Ni-NTA column. In brief, the column was preequilibrated with 100 mL binding buffer (50 mM PBS, 10 mM imidazole, pH = 8.0) at a flow rate of 0.5~1.0 mL/min. The His-tagged MS2 VLPs were bound to the columns, washed with washing buffer (100 mM PBS, 20 mM imidazole, pH = 8.0) until OD280=0, and eluted from the column using elution buffer (50 mM PBS, 250 mM imidazole, pH = 8.0) at a flow rate of 0.5 mL/min.

Ten microliters of the solution containing MS2 VLPs was dropped onto carbon-coated copper grids and then negatively stained with 2% uranyl acetate. After drying, the MS2 VLPs were observed by transmission electron microscopy (TEM) at 150,000 × magnification.

### 2.5 Determination of RNA copies in MS2 VLPs by RT–qPCR

RNA packaged into MS2 VLPs was extracted using a viral RNA extraction kit (Qmega) according to the manufacturer’s instructions and then reverse transcribed to cDNA. qPCR was performed using the following protocol: 1 cycle at 95°C for 10 min, followed by 30 cycles of 95°C for 30 seconds, 62°C for 60 seconds, and 72°C for 60 seconds. The primers and probes were synthesized by Genscript Biotech Corporation (Nanjing, China) and are listed in **Table 1**.

**Table 1:** Primer and probe sequences.

Primer	Primer sequence (5′–3′)	Size (bp)
SARS-CoV-2-F	TAAGAAGGAGATATACATATGCATATGATGTCTGATAATGGACCCC	2084
SARS-CoV-2-R	CAGCGGTGGCAGCAGCCTAGGCCTAGACATGGGTGATCCTCA	
Coat protein-F	TAAGAAGGAGATATACATATGATGGTGCGTGCGTTCAGC	2068
Coat protein -R	CAGCGGTGGCAGCAGCCTAGGTGGCCGGCGTCTATTAATAAA	
Positive control RNA-F(qPCR)	AATGAAAGATCTCAGTCCAAGATGG	287
Positive control RNA-R(qPCR)	ATTTCTTGAACTGTTGCGACTACG	
Probe(qPCR)	CCAGAAGCTGGACTTCCCTATGGTGCTAAC	

To determine the amount of contaminating DNA in the MS2 VLPs, qPCR was performed without reverse transcriptase (the enzyme was replaced with RNase-free H_2_O). qPCR was performed in duplicate for each sample.

The concentration of total nucleic acid containing RNA and DNA was calculated by the following formula:


$$\text{Concentration of total nucleic acid }(\text{copies}/\text{μL})\;=\;10^{\left(-0.3011\times \text{Ct}+12.144\right)}\times 1000$$

RNA purity was calculated by the following formula:


$$\text{RNA purity }\left(\%\right)=100\times (1-2^{\text{Ct}1-\text{Ct}2}),\text{Ct}1:\text{ Ct value in RT}-\text{qPCR},\text{ Ct}2:\text{Ct value in qPCR}$$

The concentration of RNA was calculated by the following formula:


Concentration of RNA (copies/μL) = the copy number of total nucleic acid ×RNA purity

### 2.6 Stability of RNA packaged in MS2 VLPs against ribonucleases

The purified MS2 VLPs were diluted 10,000-fold, and the stability of RNA against nucleases was determined by treatment with 10 U of RNase A at 37°C for 1 h. After incubation with RNase, RT–qPCR was performed to assess the number of RNA copies. In this assay, naked RNA extracted from MS2 VLPs was used as the control.

### 2.7 Effects of temperature on the stability of MS2 VLPs

MS2 VLPs (containing 7.85×10^10^ copies RNA/μL) were stored in PBS solution (pH 8.0) at 37°C for 90 days or -20°C for 60 weeks. Then, total RNA was extracted with a viral RNA extraction kit, and RT–qPCR was performed to determine the number of copies of RNA at different time points.

### 2.8 Effects of NaNO3-Tris buffer on the stability of MS2 VLPs

MS2 VLPs were also stored in NaNO_3_-Tris (pH 8.0) buffer at 37°C for 90 days or -20°C for 60 weeks. RT–qPCR and qPCR were performed to determine the concentration and purity of the positive control RNA, respectively.

## 3 Results

### 3.1 Construction of the recombinant plasmid pCDFDuet-MS2-SARS-CoV-2

**Figure 1:**
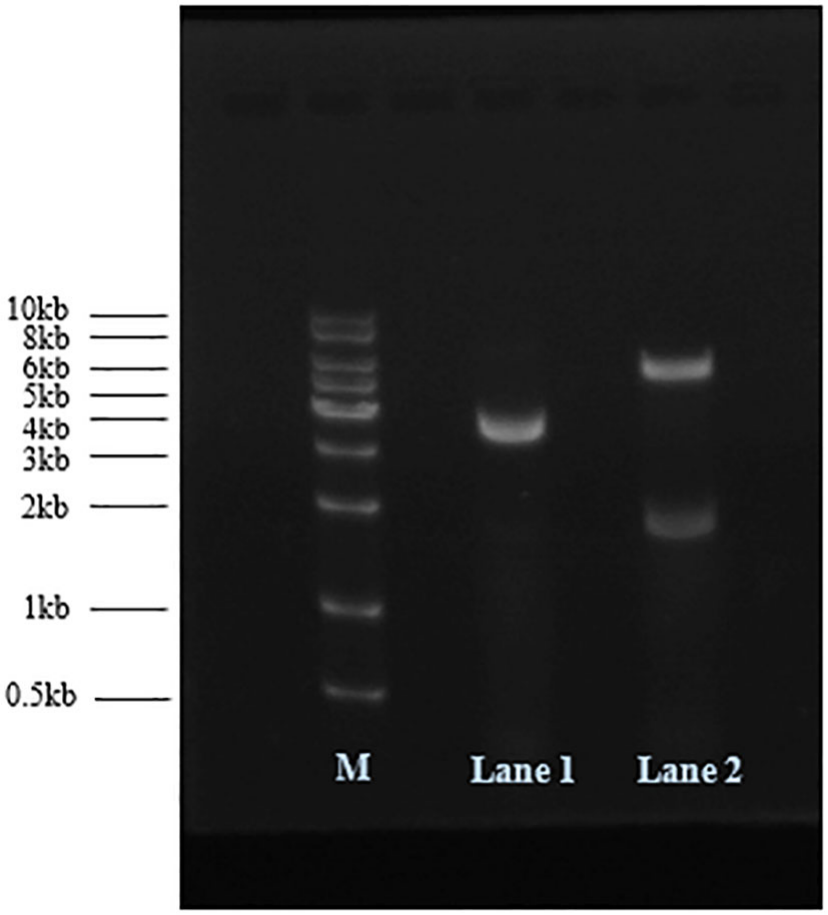
Recombinant plasmids were analyzed by 1% agarose gel electrophoresis. M: DNA marker; Lane 1: pCDFDuet-1 digested with NotI; Lane 2: pCDFDuet-MS2-SARS-CoV-2 digested with AvrII and NdeI.

The plasmid pCDFDuet-1 was linearized by NotI digestion, and pCDFDuet-MS2-SARS-CoV-2 was treated with NdeI and AvrII endonucleases at 37°C. The results of 1% agarose gel electrophoresis analysis are shown in **Figure 1**. The size of plasmid pCDFDuet-1 is 3781 bp; pCDFDuet-MS2-SARS-CoV-2 was cut by NdeI and AvrII into two fragments of 5664 bp and 1857 bp in size. The recombinant plasmid was confirmed by DNA sequencing.

### 3.2 Determination of expression of MS2 coat protein and maturase

**Figure 2:**
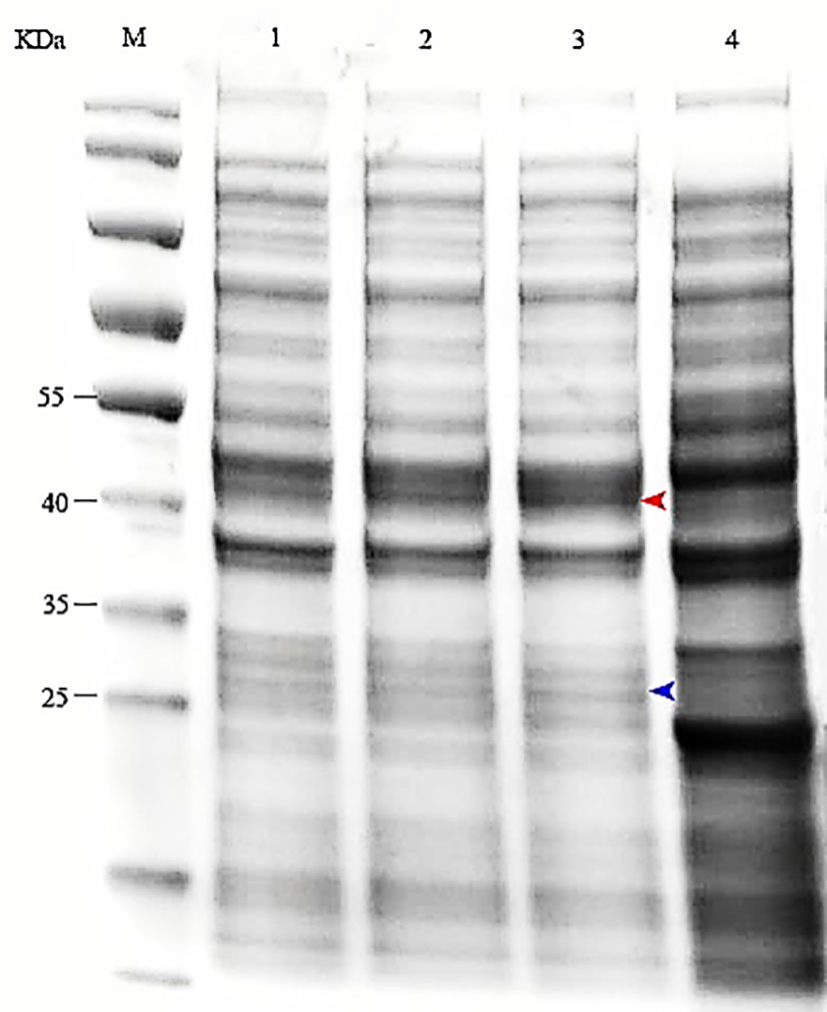
Expression of the MS2 coat protein and maturase was determined by SDS–PAGE. M: protein marker; 1-3: *E. coli* BL21(DE3) induced by 0.5, 0.8 or 1.0 mM IPTG; 4: Control (bacteria transformed with pCDFDuet-MS2-SARS-CoV-2 plasmid but uninduced).

**Figure 3:**
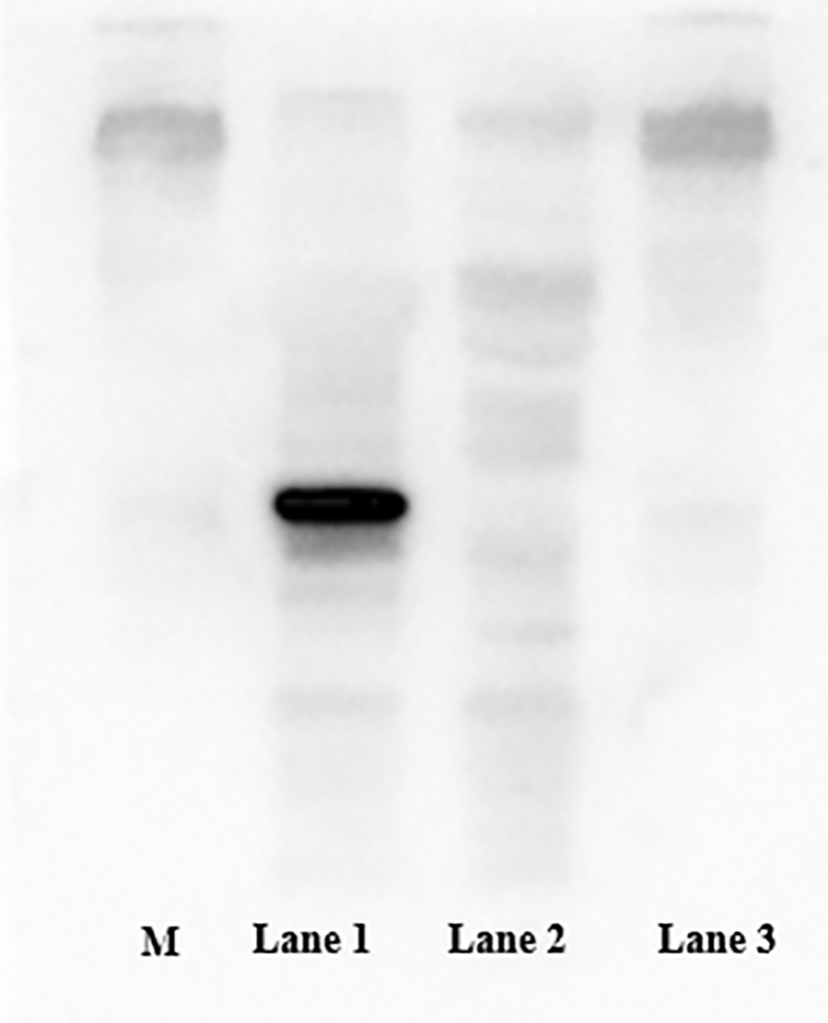
Expression of the coat protein was confirmed by western blotting using an anti-His tag antibody. M: standard protein marker; Lane 1: *E. coli* BL21(DE3) with the recombinant plasmid induced by 1.0 mM IPTG; Lane 2: *E. coli* BL21(DE3) with the recombinant plasmid not induced by IPTG; Lane 3: *E. coli* BL21(DE3) without the recombinant plasmid.

After induction by IPTG, the bacterial cells were centrifuged and the pellets collected. SDS–PAGE (12.5%) was performed to analyze expression of MS2 coat protein and maturase. The results showed that both MS2 coat protein dimer (44 kD) and maturase (29 kD) were expressed by IPTG induction in BL21 cells (**Figure 2**). In contrast, MS2 coat protein production was not observed in control *E. coli* BL21 (DE3) cells without the pCDFDuet-MS2-SARS-CoV-2 plasmid. To confirm expression of the MS2 coat protein, western blotting was performed using an anti-His-tag antibody. The results are shown in **Figure 3**.

### 3.3 Size and shape verification of MS2 VLPs

**Figure 4:**
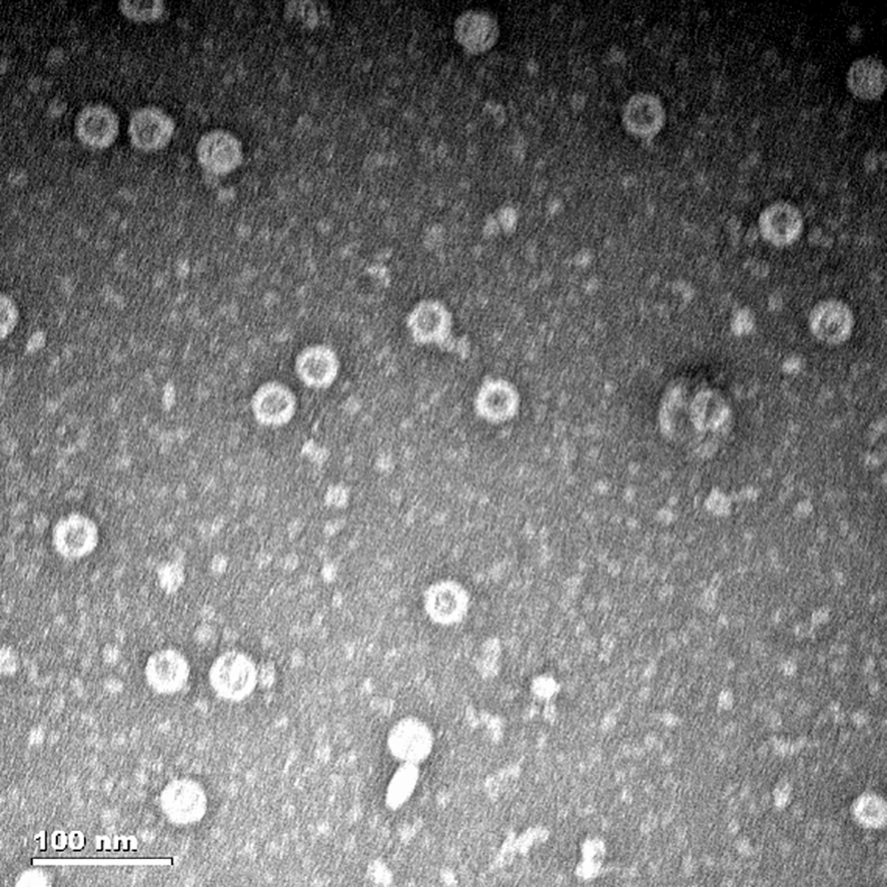
Characterization of MS2 VLPs by transmission electron microscopy (TEM). The scale bar equals 100 nm.

After purification with a Ni-NTA affinity chromatography column, the MS2 VLPs were observed by TEM. The results confirmed that the MS2 VLPs had assembled into intact capsids of approximately 30 nm in diameter. Moreover, the MS2 VLPs were not damaged by the purification process and did not form any aggregates (**Figure 4**).

### 3.4 The purity of RNA in MS2 VLPs

RT–qPCR was performed to detect the copy numbers of RNA in MS2 VLPs. The copy number of contaminating DNA in the MS2 VLPs was determined by qPCR. The results showed that the copy number of RNA in MS2 VLPs was 6.47×10^12^ copies/μL. The purity of the RNA in MS2 VLPs was 99.99%.

### 3.5 The stability of RNA in MS2 VLPs against ribonucleases

**Figure 5:**
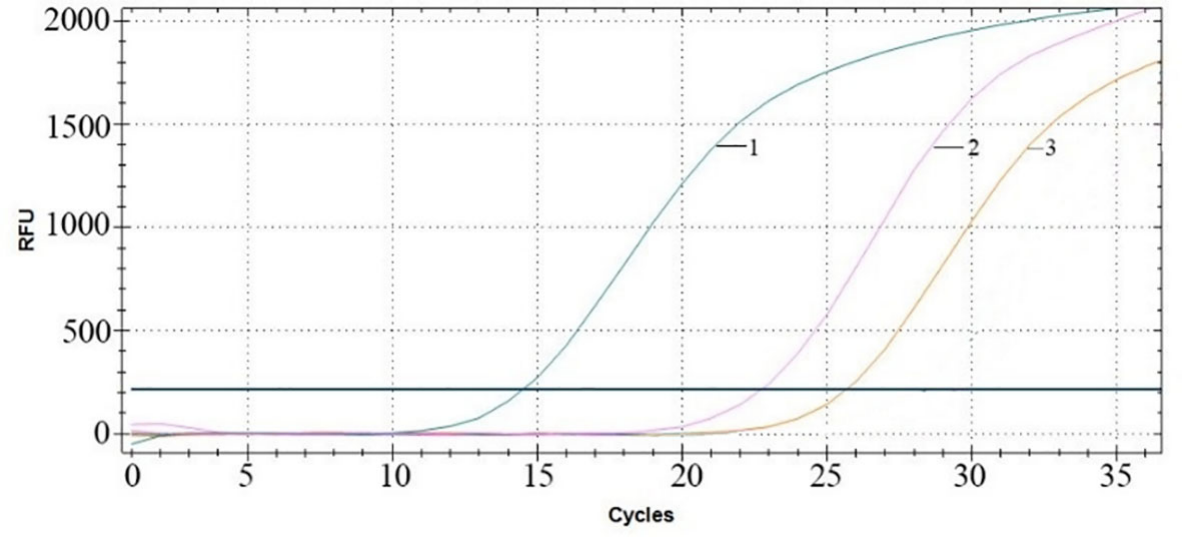
RT–qPCR amplification curves. Curve 1: MS2 VLPs in the absence of RNase. Curve 2: MS2 VLPs in the presence of RNase. Curve 3: naked RNA-extracted MS2 VLPs in the presence of RNase.

After treatment with RNase A for 1 h, RT–qPCR was performed to verify the copy numbers of RNA encapsulated in MS2 VLPs and naked RNA extracted from MS2 VLPs. The results showed 2.65×10^8^ copies/μL packaged RNA, with 2.24×10^7^ copies/μL naked RNA (**Figure 5**). These results indicate that the nanoparticles were able to effectively protect the RNA against degradation by ribonuclease.

### 3.6 Stability of MS2 VLPs at different storage temperatures

**Figure 6:**
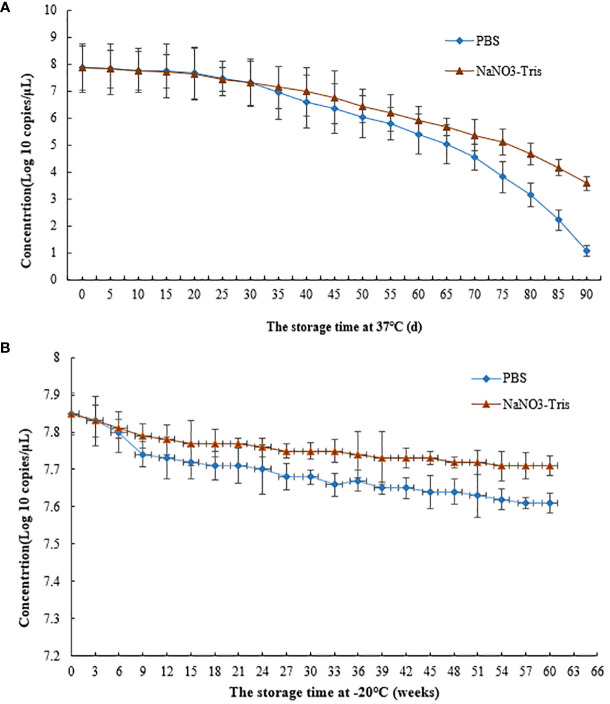
Stability of SARS-CoV-2 MS2 VLPs at 37°C and -20°C. **(A)** MS2 VLPs were stored at 37°C for 90 days. **(B)** MS2 VLPs were stored at -20°C for 60 weeks.

RT–qPCR results indicated a concentration of RNA of 7.31×10^10^ copies/μL when the VLPs were stored at 37°C for 30 days (**Figure 6A**). When the MS2 VLPs were stored at -20°C for 60 weeks, the concentration of RNA was 7.7×10^10^ copies/μL (**Figure 6B**). These results suggest that RNA packaged in the nanoparticles was stable at 37°C and -20°C. The MS2 VLPs can be used as positive control RNA, meeting the requirements for clinical testing.

Previous studies have found that the amounts of electrolytes are key factors for assembly of MS2 VLPs (Corman et al., 2020; Holshue et al., 2020; Huang et al., 2020; Wiersinga et al., 2020). Thus, the effects of NaNO3-Tris (100 mM, pH 8.0) buffer on the stability of MS2 VLPs were investigated. The results showed that NaNO3-Tris buffer significantly increased the stability of SARS-CoV-2 MS2 VLPs when the particles were stored at 37°C for 30 days or at -20°C for 9 weeks.

## 4 Discussion

MS2-based nanoparticles carrying positive RNAs can be mass-produced and have excellent characteristics as control materials for molecular diagnosis. Previous studies have shown that MS2 VLPs can serve as positive controls for molecular diagnosis of RNA viruses, such as enterovirus 71 (EV71), HIV-1, avian influenza A (H7N9), ebolavirus (EBOV), measles virus (MeV), hepatitis E virus (HEV), and dengue virus (DENV). However, the length of the exogenous RNA packaged by MS2 VLPs is limited, resulting in two or more armored RNAs being expressed for an assay of one kind of virus (Sun et al., 2013; Wang G. et al., 2015; Zhang et al., 2015; Zhang et al., 2016). The one-plasmid double-expression system allows for a maximum length of encapsidated ssRNA of 4942 nt (Prakash and Gosavi, 2021).

RT–qPCR is a rapid and accurate method for detecting SARS-CoV-2. Overall, detection should be conducted with ideal control materials, which should be stable, and the concentration of RNA should be over 10^5^ copies/μL. In this study, we prepared MS2 VLPs containing SARS-CoV-2 RNA fragments using the double expression system pCDFDuet-1. The results showed that the positive control RNA sequence (1857 nt) was successfully encapsulated into MS2 VLPs, which were stable at 37 and -20°C. These results are consistent with previous studies (Mikel et al., 2015; Fu et al., 2017).

Purification is a key procedure in MS2 VLP preparation. The traditional method to purify MS2 VLPs is ultracentrifugation, which is time-consuming and laborious. A His-tag added to the coat protein can be used for affinity purification of His-tagged MS2 VLPs (Mikel et al., 2016). Several previous studies have found that insertion of a His-tag interferes with assembly of MS2 VLPs (Luc et al., 2013; O’Rourke et al., 2015). It has also been reported that fusion of two coat protein monomers (single-chain dimer) is more tolerant to peptide insertion than coat protein monomers (Mikel et al., 2017). The pACYCDuet-1 plasmid was used to express the single-chain version of the MS2 coat protein dimer, containing one wild-type coat protein fused with one mutagenized coat protein carrying the His-tag. The His-tagged coat protein dimer and maturase successfully assembled into intact MS2 VLPs (Mikel et al., 2017). In this study, the pCDFDuet-1 system was used to express the single-chain version of the MS2 coat protein dimer fused to a His-tag. The His-tagged MS2 VLPs were efficiently prepared and purified, and the ssRNA encapsulated in the nanoparticles was resistant to RNase digestion. These VLPs displayed high stability when stored at 37°C or -20°C.

PBS, HEPES, and Tris buffers, alone or mixed with electrolytes such as MgSO_4_ or NaCl, have been chosen as protective buffers for MS2 VLP preparation and storage (Mylon et al., 2010; Dika et al., 2011; Jekhmane et al., 2017; Samuel et al., 2020). The pH value is an important factor that affects the formation and stability of VLPs (Gerba and Betancourt, 2017). It has been demonstrated that NaNO3-Tris (100 mM, pH 8.0) buffer might be helpful for formation of MS2 VLPs, substantially increasing the quality and stability of the VLPs generated (Hashemi et al., 2021). In the present study, we used NaNO_3_-Tris (100 mM, pH 8.0) buffer as the storage solution for SARS-CoV-2 MS2 VLPs, and the stability of the MS2 VLPs was obviously enhanced when stored in NaNO_3_-Tris solution.

## 5 Conclusion

SARS-CoV-2 MS2 VLPs prepared using the one-plasmid double-expression system were studied, and their stability in ribonuclease challenges or time–temperature tolerance was evaluated. The results demonstrated that SARS-CoV-2 MS2 VLPs meet the requirements for application as a positive control material in molecular diagnosis of COVID-19. In the last two decades, coronaviruses (CoVs) have caused major emerging infectious diseases, which have greatly threatened public health. To date, 7 species of CoVs have been identified as infecting humans. In the future, it is likely that high-risk CoV species will appear, jump to humans and cause widespread infection. Hence, it is essential to establish methods for rapid detection and diagnosis. One or more consensus RNA sequences from α-CoV or β-CoV can be packaged into MS2 VLPs, which may be used as the positive control of RT–qPCR assays for new high-risk CoV species.

## Data availability statement

The original contributions presented in the study are included in the article/supplementary material. Further inquiries can be directed to the corresponding author.

## Author contributions

LQ drafted the manuscript. ZZ, MW and GD performed protein expression, purification and sample preparation. ZK and HM performed the data collection and analyzed the data. JW designed the study. All authors discussed the results and commented on the manuscript. All authors contributed to the article and approved the submitted version.
